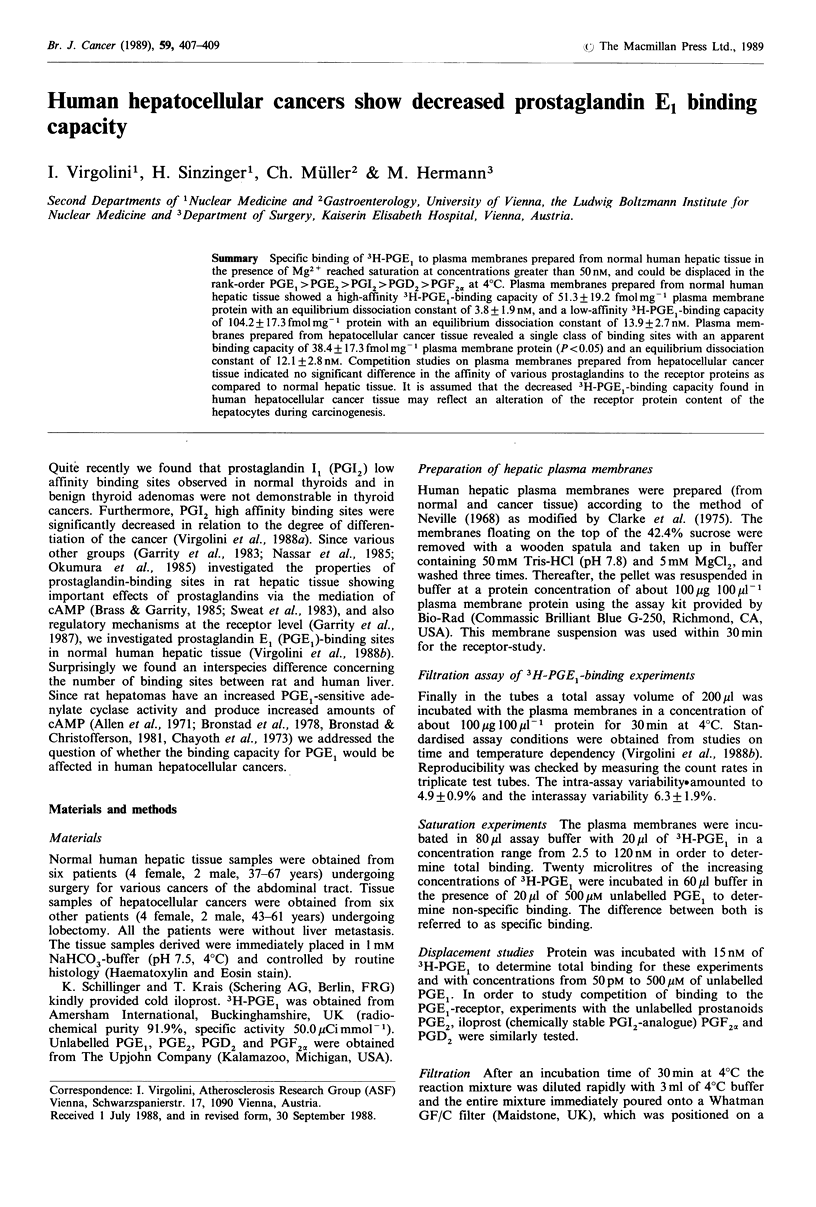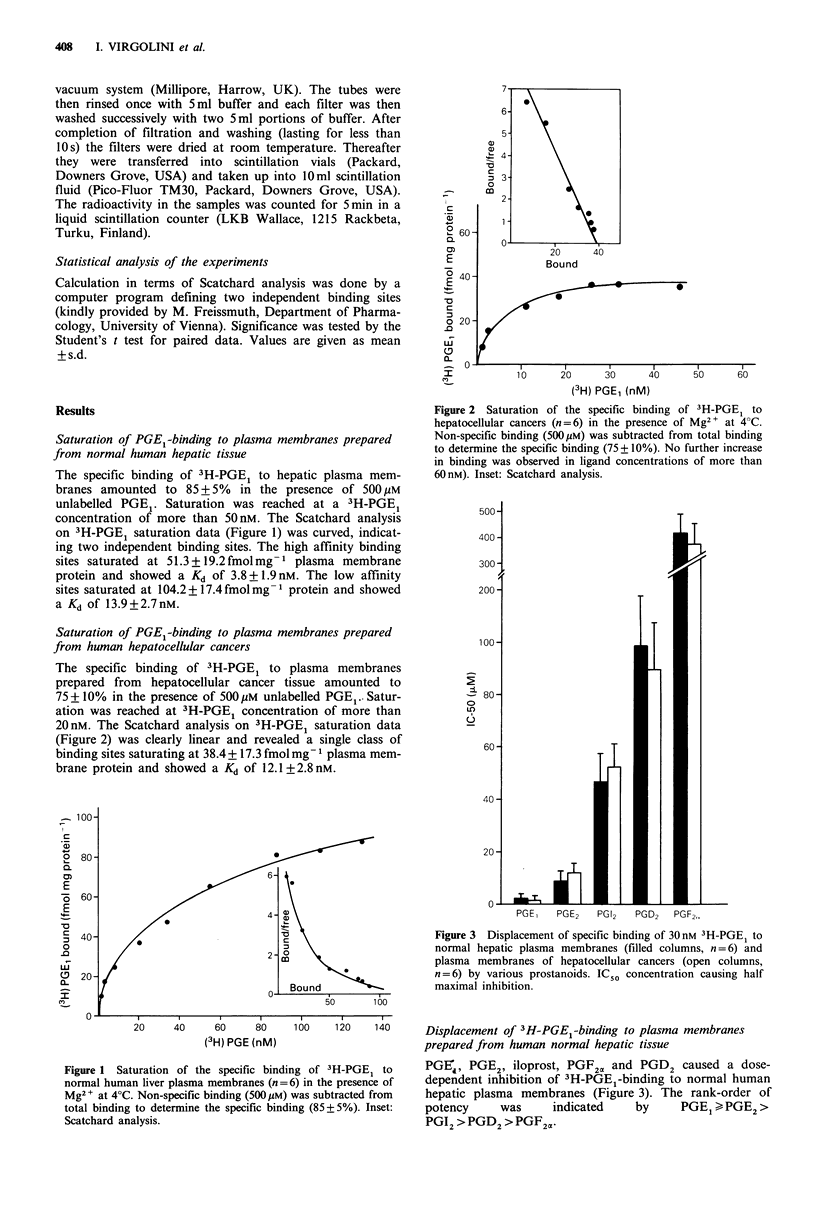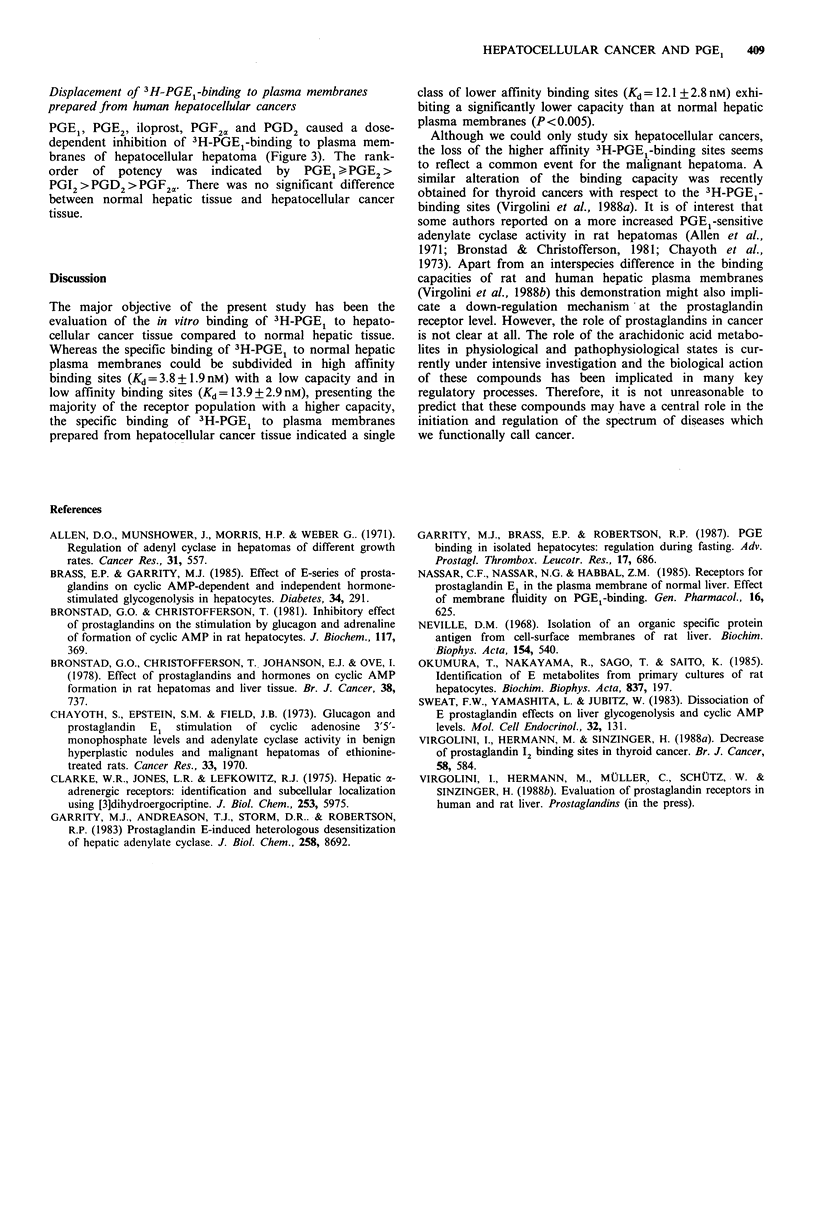# Human hepatocellular cancers show decreased prostaglandin E1 binding capacity.

**DOI:** 10.1038/bjc.1989.81

**Published:** 1989-03

**Authors:** I. Virgolini, H. Sinzinger, C. MÃ¼ller, M. Hermann

**Affiliations:** Second Department of Nuclear Medicine, Ludwig Boltzmann Institute for Nuclear Medicine, Austria.

## Abstract

Specific binding of 3H-PGE1 to plasma membranes prepared from normal human hepatic tissue in the presence of Mg2+ reached saturation at concentrations greater than 50 nM, and could be displaced in the rank-order PGE1 greater than PGE2 greater than PGI2 greater than PGD2 greater than PGF2 alpha at 4 degrees C. Plasma membranes prepared from normal human hepatic tissue showed a high-affinity 3H-PGE1-binding capacity of 51.3 +/- 19.2 fmol mg-1 plasma membrane protein with an equilibrium dissociation constant of 3.8 +/- 1.9 nM, and a low-affinity 3H-PGE1-binding capacity of 104.2 +/- 17.3 fmol mg-1 protein with an equilibrium dissociation constant of 13.9 +/- 2.7 nM. Plasma membranes prepared from hepatocellular cancer tissue revealed a single class of binding sites with an apparent binding capacity of 38.4 +/- 17.3 fmol mg-1 plasma membrane protein (P less than 0.05) and an equilibrium dissociation constant of 12.1 +/- 2.8 nM. Competition studies on plasma membranes prepared from hepatocellular cancer tissue indicated no significant difference in the affinity of various prostaglandins to the receptor proteins as compared to normal hepatic tissue. It is assumed that the decreased 3H-PGE1-binding capacity found in human hepatocellular cancer tissue may reflect an alteration of the receptor protein content of the hepatocytes during carcinogenesis.


*Br. J. Cancer* (1989), **59**, 407–409

© The Macmillan Press Ltd., 1989

### Human hepatocellular cancers show decreased prostaglandin $E_1$ binding capacity

I. Virgolini[1], H. Sinzinger[1], Ch. Müller[2] & M. Hermann[3]

*Second Departments of [1]Nuclear Medicine and [2]Gastroenterology, University of Vienna, the Ludwig Boltzmann Institute for Nuclear Medicine and [3]Department of Surgery, Kaiserin Elisabeth Hospital, Vienna, Austria.*

**Summary** Specific binding of $^3H$-$PGE_1$ to plasma membranes prepared from normal human hepatic tissue in the presence of $Mg^{2+}$ reached saturation at concentrations greater than 50 nM, and could be displaced in the rank-order $PGE_1 > PGE_2 > PGI_2 > PGD_2 > PGF_{2\alpha}$ at 4°C. Plasma membranes prepared from normal human hepatic tissue showed a high-affinity $^3H$-$PGE_1$-binding capacity of $51.3 \pm 19.2$ fmol mg$^{-1}$ plasma membrane protein with an equilibrium dissociation constant of $3.8 \pm 1.9$ nM, and a low-affinity $^3H$-$PGE_1$-binding capacity of $104.2 \pm 17.3$ fmol mg$^{-1}$ protein with an equilibrium dissociation constant of $13.9 \pm 2.7$ nM. Plasma membranes prepared from hepatocellular cancer tissue revealed a single class of binding sites with an apparent binding capacity of $38.4 \pm 17.3$ fmol mg$^{-1}$ plasma membrane protein ($P < 0.05$) and an equilibrium dissociation constant of $12.1 \pm 2.8$ nM. Competition studies on plasma membranes prepared from hepatocellular cancer tissue indicated no significant difference in the affinity of various prostaglandins to the receptor proteins as compared to normal hepatic tissue. It is assumed that the decreased $^3H$-$PGE_1$-binding capacity found in human hepatocellular cancer tissue may reflect an alteration of the receptor protein content of the hepatocytes during carcinogenesis.

Quite recently we found that prostaglandin $I_1$ ($PGI_2$) low affinity binding sites observed in normal thyroids and in benign thyroid adenomas were not demonstrable in thyroid cancers. Furthermore, $PGI_2$ high affinity binding sites were significantly decreased in relation to the degree of differentiation of the cancer (Virgolini *et al.*, 1988*a*). Since various other groups (Garrity *et al.*, 1983; Nassar *et al.*, 1985; Okumura *et al.*, 1985) investigated the properties of prostaglandin-binding sites in rat hepatic tissue showing important effects of prostaglandins via the mediation of cAMP (Brass & Garrity, 1985; Sweat *et al.*, 1983), and also regulatory mechanisms at the receptor level (Garrity *et al.*, 1987), we investigated prostaglandin $E_1$ ($PGE_1$)-binding sites in normal human hepatic tissue (Virgolini *et al.*, 1988*b*). Surprisingly we found an interspecies difference concerning the number of binding sites between rat and human liver. Since rat hepatomas have an increased $PGE_1$-sensitive adenylate cyclase activity and produce increased amounts of cAMP (Allen *et al.*, 1971; Bronstad *et al.*, 1978, Bronstad & Christofferson, 1981, Chayoth *et al.*, 1973) we addressed the question of whether the binding capacity for $PGE_1$ would be affected in human hepatocellular cancers.

### Materials and methods

### *Materials*

Normal human hepatic tissue samples were obtained from six patients (4 female, 2 male, 37–67 years) undergoing surgery for various cancers of the abdominal tract. Tissue samples of hepatocellular cancers were obtained from six other patients (4 female, 2 male, 43–61 years) undergoing lobectomy. All the patients were without liver metastasis. The tissue samples derived were immediately placed in 1 mM $NaHCO_3$-buffer (pH 7.5, 4°C) and controlled by routine histology (Haematoxylin and Eosin stain).

K. Schillinger and T. Krais (Schering AG, Berlin, FRG) kindly provided cold iloprost. $^3H$-$PGE_1$ was obtained from Amersham International, Buckinghamshire, UK (radiochemical purity 91.9%, specific activity 50.0 $\mu$Ci mmol$^{-1}$). Unlabelled $PGE_1$, $PGE_2$, $PGD_2$ and $PGF_{2\alpha}$ were obtained from The Upjohn Company (Kalamazoo, Michigan, USA).

Correspondence: I. Virgolini, Atherosclerosis Research Group (ASF) Vienna, Schwarzspanierstr. 17, 1090 Vienna, Austria.
Received 1 July 1988, and in revised form, 30 September 1988.

### *Preparation of hepatic plasma membranes*

Human hepatic plasma membranes were prepared (from normal and cancer tissue) according to the method of Neville (1968) as modified by Clarke *et al.* (1975). The membranes floating on the top of the 42.4% sucrose were removed with a wooden spatula and taken up in buffer containing 50 mM Tris-HCl (pH 7.8) and 5 mM $MgCl_2$, and washed three times. Thereafter, the pellet was resuspended in buffer at a protein concentration of about 100 $\mu$g 100 $\mu$l$^{-1}$ plasma membrane protein using the assay kit provided by Bio-Rad (Commassic Brilliant Blue G-250, Richmond, CA, USA). This membrane suspension was used within 30 min for the receptor-study.

### *Filtration assay of $^3H$-$PGE_1$-binding experiments*

Finally in the tubes a total assay volume of 200 $\mu$l was incubated with the plasma membranes in a concentration of about 100 $\mu$g 100 $\mu$l$^{-1}$ protein for 30 min at 4°C. Standardised assay conditions were obtained from studies on time and temperature dependency (Virgolini *et al.*, 1988*b*). Reproducibility was checked by measuring the count rates in triplicate test tubes. The intra-assay variability amounted to $4.9 \pm 0.9$% and the interassay variability $6.3 \pm 1.9$%.

*Saturation experiments* The plasma membranes were incubated in 80 $\mu$l assay buffer with 20 $\mu$l of $^3H$-$PGE_1$ in a concentration range from 2.5 to 120 nM in order to determine total binding. Twenty microlitres of the increasing concentrations of $^3H$-$PGE_1$ were incubated in 60 $\mu$l buffer in the presence of 20 $\mu$l of 500 $\mu$M unlabelled $PGE_1$ to determine non-specific binding. The difference between both is referred to as specific binding.

*Displacement studies* Protein was incubated with 15 nM of $^3H$-$PGE_1$ to determine total binding for these experiments and with concentrations from 50 pM to 500 $\mu$M of unlabelled $PGE_1$. In order to study competition of binding to the $PGE_1$-receptor, experiments with the unlabelled prostanoids $PGE_2$, iloprost (chemically stable $PGI_2$-analogue) $PGF_{2\alpha}$ and $PGD_2$ were similarly tested.

*Filtration* After an incubation time of 30 min at 4°C the reaction mixture was diluted rapidly with 3 ml of 4°C buffer and the entire mixture immediately poured onto a Whatman GF/C filter (Maidstone, UK), which was positioned on a vacuum system (Millipore, Harrow, UK). The tubes were then rinsed once with 5 ml buffer and each filter was then washed successively with two 5 ml portions of buffer. After completion of filtration and washing (lasting for less than 10 s) the filters were dried at room temperature. Thereafter they were transferred into scintillation vials (Packard, Downers Grove, USA) and taken up into 10 ml scintillation fluid (Pico-Fluor TM30, Packard, Downers Grove, USA). The radioactivity in the samples was counted for 5 min in a liquid scintillation counter (LKB Wallace, 1215 Rackbeta, Turku, Finland).

*Statistical analysis of the experiments*

Calculation in terms of Scatchard analysis was done by a computer program defining two independent binding sites (kindly provided by M. Freissmuth, Department of Pharmacology, University of Vienna). Significance was tested by the Student's *t* test for paired data. Values are given as mean ±s.d.

### Results

*Saturation of $PGE_1$-binding to plasma membranes prepared from normal human hepatic tissue*

The specific binding of $^3H$-$PGE_1$ to hepatic plasma membranes amounted to 85±5% in the presence of 500 μM unlabelled $PGE_1$. Saturation was reached at a $^3H$-$PGE_1$ concentration of more than 50 nM. The Scatchard analysis on $^3H$-$PGE_1$ saturation data (Figure 1) was curved, indicating two independent binding sites. The high affinity binding sites saturated at 51.3±19.2 fmol $mg^{-1}$ plasma membrane protein and showed a $K_d$ of 3.8±1.9 nM. The low affinity sites saturated at 104.2±17.4 fmol $mg^{-1}$ protein and showed a $K_d$ of 13.9±2.7 nM.

*Saturation of $PGE_1$-binding to plasma membranes prepared from human hepatocellular cancers*

The specific binding of $^3H$-$PGE_1$ to plasma membranes prepared from hepatocellular cancer tissue amounted to 75±10% in the presence of 500 μM unlabelled $PGE_1$. Saturation was reached at $^3H$-$PGE_1$ concentration of more than 20 nM. The Scatchard analysis on $^3H$-$PGE_1$ saturation data (Figure 2) was clearly linear and revealed a single class of binding sites saturating at 38.4±17.3 fmol $mg^{-1}$ plasma membrane protein and showed a $K_d$ of 12.1±2.8 nM.

**Figure 1** Saturation of the specific binding of $^3H$-$PGE_1$ to normal human liver plasma membranes ($n=6$) in the presence of $Mg^{2+}$ at 4°C. Non-specific binding (500 μM) was subtracted from total binding to determine the specific binding (85±5%). Inset: Scatchard analysis.

**Figure 2** Saturation of the specific binding of $^3H$-$PGE_1$ to hepatocellular cancers ($n=6$) in the presence of $Mg^{2+}$ at 4°C. Non-specific binding (500 μM) was subtracted from total binding to determine the specific binding (75±10%). No further increase in binding was observed in ligand concentrations of more than 60 nM). Inset: Scatchard analysis.

**Figure 3** Displacement of specific binding of 30 nM $^3H$-$PGE_1$ to normal hepatic plasma membranes (filled columns, $n=6$) and plasma membranes of hepatocellular cancers (open columns, $n=6$) by various prostanoids. $IC_{50}$ concentration causing half maximal inhibition.

*Displacement of $^3H$-$PGE_1$-binding to plasma membranes prepared from human normal hepatic tissue*

$PGE_1$, $PGE_2$, iloprost, $PGF_{2\alpha}$ and $PGD_2$ caused a dose-dependent inhibition of $^3H$-$PGE_1$-binding to normal human hepatic plasma membranes (Figure 3). The rank-order of potency was indicated by $PGE_1 \geq PGE_2 > PGI_2 > PGD_2 > PGF_{2\alpha}$.

*Displacement of $^3H$-$PGE_1$-binding to plasma membranes prepared from human hepatocellular cancers*

$PGE_1$, $PGE_2$, iloprost, $PGF_{2\alpha}$ and $PGD_2$ caused a dose-dependent inhibition of $^3H$-$PGE_1$-binding to plasma membranes of hepatocellular hepatoma (Figure 3). The rank-order of potency was indicated by $PGE_1 \geqslant PGE_2 > PGI_2 > PGD_2 > PGF_{2\alpha}$. There was no significant difference between normal hepatic tissue and hepatocellular cancer tissue.

### Discussion

The major objective of the present study has been the evaluation of the *in vitro* binding of $^3H$-$PGE_1$ to hepatocellular cancer tissue compared to normal hepatic tissue. Whereas the specific binding of $^3H$-$PGE_1$ to normal hepatic plasma membranes could be subdivided in high affinity binding sites ($K_d = 3.8 \pm 1.9$ nM) with a low capacity and in low affinity binding sites ($K_d = 13.9 \pm 2.9$ nM), presenting the majority of the receptor population with a higher capacity, the specific binding of $^3H$-$PGE_1$ to plasma membranes prepared from hepatocellular cancer tissue indicated a single class of lower affinity binding sites ($K_d = 12.1 \pm 2.8$ nM) exhibiting a significantly lower capacity than at normal hepatic plasma membranes ($P < 0.005$).

Although we could only study six hepatocellular cancers, the loss of the higher affinity $^3H$-$PGE_1$-binding sites seems to reflect a common event for the malignant hepatoma. A similar alteration of the binding capacity was recently obtained for thyroid cancers with respect to the $^3H$-$PGE_1$-binding sites (Virgolini *et al.*, 1988*a*). It is of interest that some authors reported on a more increased $PGE_1$-sensitive adenylate cyclase activity in rat hepatomas (Allen *et al.*, 1971; Bronstad & Christofferson, 1981; Chayoth *et al.*, 1973). Apart from an interspecies difference in the binding capacities of rat and human hepatic plasma membranes (Virgolini *et al.*, 1988*b*) this demonstration might also implicate a down-regulation mechanism at the prostaglandin receptor level. However, the role of prostaglandins in cancer is not clear at all. The role of the arachidonic acid metabolites in physiological and pathophysiological states is currently under intensive investigation and the biological action of these compounds has been implicated in many key regulatory processes. Therefore, it is not unreasonable to predict that these compounds may have a central role in the initiation and regulation of the spectrum of diseases which we functionally call cancer.

### References

ALLEN, D.O., MUNSHOWER, J., MORRIS, H.P. & WEBER G.. (1971). Regulation of adenyl cyclase in hepatomas of different growth rates. *Cancer Res.*, **31**, 557.

BRASS, E.P. & GARRITY, M.J. (1985). Effect of E-series of prostaglandins on cyclic AMP-dependent and independent hormone-stimulated glycogenolysis in hepatocytes. *Diabetes*, **34**, 291.

BRONSTAD, G.O. & CHRISTOFFERSON, T. (1981). Inhibitory effect of prostaglandins on the stimulation by glucagon and adrenaline of formation of cyclic AMP in rat hepatocytes. *J. Biochem.*, **117**, 369.

BRONSTAD, G.O., CHRISTOFFERSON, T.. JOHANSON, E.J. & OVE, I. (1978). Effect of prostaglandins and hormones on cyclic AMP formation in rat hepatomas and liver tissue. *Br. J. Cancer*, **38**, 737.

CHAYOTH, S., EPSTEIN, S.M. & FIELD, J.B. (1973). Glucagon and prostaglandin $E_1$ stimulation of cyclic adenosine 3′5′-monophosphate levels and adenylate cyclase activity in benign hyperplastic nodules and malignant hepatomas of ethionine-treated rats. *Cancer Res.*, **33**, 1970.

CLARKE, W.R., JONES, L.R. & LEFKOWITZ, R.J. (1975). Hepatic α-adrenergic receptors: identification and subcellular localization using [3]dihydroergocriptine. *J. Biol. Chem.*, **253**, 5975.

GARRITY, M.J., ANDREASON, T.J., STORM, D.R.. & ROBERTSON, R.P. (1983) Prostaglandin E-induced heterologous desensitization of hepatic adenylate cyclase. *J. Biol. Chem.*, **258**, 8692.

GARRITY, M.J., BRASS, E.P. & ROBERTSON, R.P. (1987). PGE binding in isolated hepatocytes: regulation during fasting. *Adv. Prostagl. Thrombox. Leucotr. Res.*, **17**, 686.

NASSAR, C.F., NASSAR, N.G. & HABBAL, Z.M. (1985). Receptors for prostaglandin $E_1$ in the plasma membrane of normal liver. Effect of membrane fluidity on $PGE_1$-binding. *Gen. Pharmacol.*, **16**, 625.

NEVILLE, D.M. (1968). Isolation of an organic specific protein antigen from cell-surface membranes of rat liver. *Biochim. Biophys. Acta*, **154**, 540.

OKUMURA, T., NAKAYAMA, R., SAGO, T. & SAITO, K. (1985). Identification of E metabolites from primary cultures of rat hepatocytes. *Biochim. Biophys. Acta*, **837**, 197.

SWEAT, F.W., YAMASHITA, L. & JUBITZ, W. (1983). Dissociation of E prostaglandin effects on liver glycogenolysis and cyclic AMP levels. *Mol. Cell Endocrinol.*, **32**, 131.

VIRGOLINI, I., HERMANN, M. & SINZINGER, H. (1988*a*). Decrease of prostaglandin $I_2$ binding sites in thyroid cancer. *Br. J. Cancer*, **58**, 584.

VIRGOLINI, I., HERMANN, M., MÜLLER, C., SCHÜTZ, W. & SINZINGER, H. (1988*b*). Evaluation of prostaglandin receptors in human and rat liver. *Prostaglandins* (in the press).